# Third birth intention of the childbearing-age population in mainland China and sociodemographic differences: a cross-sectional survey

**DOI:** 10.1186/s12889-021-12338-8

**Published:** 2021-12-14

**Authors:** Zhang Yan, Lin Hui, Jiang Wenbin, Lu Liuxue, Li Yuemei, Lv Bohan, Wei Lili

**Affiliations:** 1 Faculty, Department of Nursing, The Affiliated Hospital of Qingdao University, Qingdao, 266003 China; 2 Department of Critical Care Medicine, The Affiliated Hospital of Qingdao University, Qingdao, 266003 China; 3 Department of Nursing and Hospital Infection Management, The Affiliated Hospital of Qingdao University, Qingdao, 266003 China; 4 Department of Nursing, Affiliated Hospital of Youjiang Medical University for Nationalities, Baise, 533000 China; 5 Department of Nursing, Qinghai Provincial People’s Hospital, Xining, 810007 China; 6 School of Nursing, Qingdao University, Qingdao, 266071 China; 7 Department of Nursing, The Affiliated Hospital of Qingdao University, 16# Jiangsu Road, Qingdao, 266003 Shandong China

**Keywords:** Third birth intention, Childbearing-age, Influencing factors

## Abstract

**Background:**

Global fertility declines have become an inevitable trend, and many countries are adopting policies to drive fertility increases. Fertility intention plays an important role in predicting fertility behavior. The Chinese government has recently issued the ‘three-child’ policy, and there is still little research on the third birth intention of the childbearing-age population. Therefore, the aim of this study is to investigate the prevalence and related reasons of third birth intention in the childbearing-age population in mainland China, and analyze the sociodemographic differences.

**Method:**

A cross-sectional survey was conducted in mainland China from June to July 2021. A total of 15,332 childbearing-age participants responded and completed the Fertility Intention Questionnaire online through the Wenjuanxing Platform. Data were explored and analyzed by SPSS (version 22.0) software. Descriptive statistics were used to describe the current situation and reasons of third birth intention. Binary logistic regression analysis was applied to assess the influencing factors in the sociodemographic level.

**Results:**

The mean age of the participants was 32.9 ± 5.94 years. Only 12.2% of participants reported having third birth intention. The subjective norm of having both son and daughter (22.0%) and busy at work (29.2%) accounted for the largest proportion in the reasons of acceptance and rejection, respectively. Age has negative impact on third birth intention (*OR* = 0.960). Men were 2.209 times more likely to have three children than women (*P* < 0.001). With the improvement of education and family monthly income, the birth intention shows a downward trend. Compared with Han nationalities, first marriage and city residents, the ethnic minorities, remarriage and rural residents have stronger birth intention (all *P* < 0.05). And individuals with two existing children are inclined to have the third child (*OR* = 1.839).

**Conclusion:**

The third birth intention in the childbearing-age population in China is still low after the announcement of the three-child policy. It is necessary to create a favorable fertility context for childbearing-age group with high level of third birth intention, like younger, male, minority, remarriage, with lower education and family monthly income, living in rural and two existing children. Furthermore, removing barriers for those unintended is also prominent to ensure the impetus of policy.

**Supplementary Information:**

The online version contains supplementary material available at 10.1186/s12889-021-12338-8.

Worldwide, total fertility rates are decreasing, contributing to a reduction in the population sizes of many countries. According to the World Fertility and Family Planning 2020 document issued by the United Nations, the global fertility rate declined from 3.2 live births per woman in 1990 to 2.5 in 2019 [1]. Furthermore, the level of fertility is expected to continue declining. The global level of fertility is expected to reach 2.2 live births per woman in 2050 and 1.9 in 2100, according to the United Nations medium-variant projection [1]. Other censuses have made similar observations. As reported in the Iranian population census, the overall fertility rate of the Iranian population gradually decreased to 1.69 children per woman in 2015 and then to 1.61 children per woman in 2018 [2]. According to data from the Seventh National Census, the total fertility rate in China reached a low of 1.3 in 2020, and China has already fallen into the low fertility trap [3]. In Denmark, the average fertility rate in the past 40 years has been approximately 1.7 children per woman [4]. Countries such as Germany and Japan have already seen a decline of approximately 50% in the number of young adults and children. As a result, birth rates have been below the replacement level of 2.1 children per woman [5].

The falling birth rate will lead to a decline in the labor force, economic development restrictions, insufficient innovation and population aging, especially in highly industrialized countries [4]. A global aging society poses severe economic, healthcare and humanitarian challenges [6]. There has been a dramatic surge in the number of older adults diagnosed with neurodegenerative diseases, leading to rigid spending on pensions and aggravating the societal burden associated with healthcare management [7]. In recent years, all of these issues have attracted widespread attention from the international community.

Generally, many countries embrace family planning as one of several methods to decrease the negative impacts of demographic transition. For example, the Chinese government issued the conditional ‘two-child’ policy in November 2013, permitting couples in which at least one of partner was an only child to have two children in order to phase out the one-child policy and reverse the low fertility rate [8]. However, the policy faltered, and the fertility potential released by policy adjustment was far from expected. In October 2015, the government issued the ‘universal second child’ policy to stimulate fertility, under which couples could have two children without the only child limitation. Then, the fertility rate increased by 12.95‰ and 12.43‰ in 2016 and 2017, respectively [3]. However, the follow-up impetus of the policy was insufficient and played a limited role in boosting fertility behavior. The birth rates in 2018 and 2019 decreased by 1.17‰ and 1.63‰, compared with the 2011–2015 period [3]. On May 31, 2021, the Political Bureau of the CPC Central Committee announced that ‘a couple can have three children and enjoy supporting measures’ [9]. Whether the issuance of this policy will reverse the status quo of low fertility rates and contribute to delaying the process of population aging remains to be seen.

The improvement of married individuals’ ability to have the desired number of children is a major challenge, and the implementation of fertility programs requires consideration of the fertility intention of couples and the elimination of barriers [10]. Recently, research on fertility intention has become the core of fertility discussion because of its unreplaceable position in the mediation of fertility behavior [11]. However, only a few studies have been conducted to evaluate fertility intention in the general population [2]. Recent surveys on childbirth intentions have mostly concentrated on special groups, such as AIDS patients, adolescents, and cancer patients [12–14]. All of these were specific in terms of basic physical condition. There are still few studies on the main driver of fertility, the general population, making it difficult to interpret the choices made by those with potential fertility compromise. Moreover, few studies have conducted surveys on the willingness to reproduce when a policy is released. The aim of this study is to evaluate the third birth intention of the general population in China after the announcement of the three-child policy, identify the reasons behind participants’ decision regarding whether to have three children, and analyze influencing factors in the aspect of sociodemographic characteristics.

Birth intention could be defined as a mental mechanism that is used to explain behaviors by portraying the individual as an actor who has desires and who attempts to achieve the pregnancy, which is directed by her beliefs [15, 16]. In the current study, we conceptualize third birth intention as the desire or intent to have three children. Fertility intentions are influenced by many cultural, demographic, health, and psychosocial factors [17]. A review indicated that having completed an education, holding a job, and a stable income and house ownership are prominent for their decision-making of childbearing [18]. The age and the number of children they already had are also particularly important factors which accounted for more difference between individuals’ fertility intention [19]. The analysis of influencing factors in this study is limited to general demographic characteristics which are relatively constant and could help identify potential groups willing to have a third child. Unlike previous studies, this study also evaluated the wishes of their spouses instead of focusing on only the husband or wife. Even we did not examine the impact of announcement of the three-child policy on third birth intention, it is expected that the evidence generated from the findings will be useful for family planning policy targeting the most relevant population in China.

## Methods

### Design

A cross-sectional survey was conducted in mainland China during a one-month period from June 10th to July 12th, 2021.

### Sample and setting

A convenience sampling method was used to recruit participants. People of childbearing age who lived in mainland China, understood Chinese, aged between 20 years and 45 years old with informed consent and voluntary participation were eligible for the study. And those unmarried or pregnant of the third child at the time of survey were excluded. We used WeChat as the main method to publish recruitment announcements for population selection. Wenjuanxing was used for questionnaire dissemination because it is the most popular social media platform in China, with 1.15 billion active users [20]. A total of 15,385 participants finished the questionnaires, of whom 12 were from overseas or not from mainland China, and 41 invalid data with anomalies or contradictions, were excluded. Finally, 15,332 participants were included in the analysis.

### Questionnaires

#### Sociodemographic characteristics

A sociodemographic questionnaire prepared by the research team was used for data collection. It collected information on age, gender, marital status (first marriage or remarriage), ethnic group (Han or Minority), education level, occupation, family monthly income, residence (city or rural), geographical region, house ownership and number of existing children. All of those individual’s background characteristics were external factors influencing fertility intentions [21].

#### Fertility intention questionnaire

Three-child fertility intention, as the dependent variable and primary outcome, was measured first by one question ‘Do you intend to have three children?’ with the response options being ‘very unintended, unintended, intended, slightly intended, and strongly intended’ accordingly. The third birth intention of their spouses are reported by their husbands or wives who participated in this research, because a fertility report of the spouse themself is not easy to acquire. And the responses are divided into four categories, including uncertain, equal intention, stronger intention of husband, and stronger intention of wife.

According to the Theory of Planned Behaviour, the intention to have or not have a child could be explained by three determinants, attitudes, subjective norm and perceived control [21]. The reasons of third birth intention were determined based on the rationale and China’s cultural context, consisting of 11 acceptance factors and 11 rejection factors. The acceptance reasons include being conducive to the growth of children, purely like children, more children bring more blessings, reducing pension risks, enhancing couple’s relationship, having both son and daughter, elders’ expectations, husband/wife expectations, influence of surrounding peer groups, health status of existing children and increasing family labor. And the rejection reasons are composed of husband/wife does not want, elders don’t want, the first or second child does not want, impact of the concept of fewer births, older ages, busy at work, having no time and energy to take care of children, high cost of upbringing and education of children, more fertility impairs health, pursuit personal career development, fear of childbirth pain and worried about body shape.

### Data collection

Wenjuanxing (www.wjx.cn), the first and largest domestic online questionnaire survey and test platform, released 125 million questionnaires and collected 9.923 billion questionnaires from respondents until July 2021. A standardized set of instructions was compiled by the researchers, including information on the purpose and significance of this study. The electronic questionnaires with formatted instructions were sent to the participants who met the inclusion criteria via WeChat groups. Participants were informed that their participation was anonymous and voluntary. To ensure the effectiveness of data collection, all survey items needed to be answered before the form could be submitted. In addition, the provided data were screened in advance. If the entered age did not meet the inclusion criteria, a prompt would indicate ineligible data. Participants were assigned identification numbers, each of which could be used only once. After data collection, the questionnaire responses were collated and examined.

### Data analysis

The data collected in Wenjuanxing were exported into Excel in text form. After preprocess of screening and assignment, the data were imported into SPSS 22.0 for statistical analysis. We used descriptive analysis to illustrate the birth intention and sociodemographic characteristics of the total participants, female group and male group separately. The reasons of intended or unintended to have the third child were described by response frequency. The third birth intention was a kind of orderly and multi-categorized data, but the *p* value of parallel lines test was less than 0.001 and the ordinal logistic regression was not suitable. Then, the dependent variable, third birth intention, was labeled ‘unintended’ (very unintended/unintended) or ‘intended’ (intended/slightly intended/strongly intended). A linear regression model was run for collinearity diagnosis, and then the binary regression analysis was conducted. Except for the age, all the other variables are considered as categorical variables. The first set of categorical variables was selected as a reference, and continuous variables were directly included in the equation. The selection of independent variables affecting third birth intention was identified by the likelihood ratio test of forward maximum (partial) likelihood estimation. The Hosmer and Lemeshow test was used to determine the goodness-of-fit of the model. Statistical significance was defined as a two-sided *p* value less than 0.05.

### Ethics approval and consent to participate

Ethical approval was obtained from Medical Ethics Committee of Affiliated Hospital of Qingdao University, who agreed with all experimental protocols. A cover letter was presented and all respondents were informed with explanation of the study aims and procedures. Their agreement to participate was asserted by choosing the ‘I agree’ option ahead of filling in the questionnairs, which help assure that all the respondents have agreed to participant in this survey. The completed questionnaires were kept confidential using file encryption software and were accessible only to the study authors responsible for data analysis. All methods were carried out in accordance with STROBE Statement.

## Results

### Participants’ sociodemographic characteristics

**Fig. 1:**
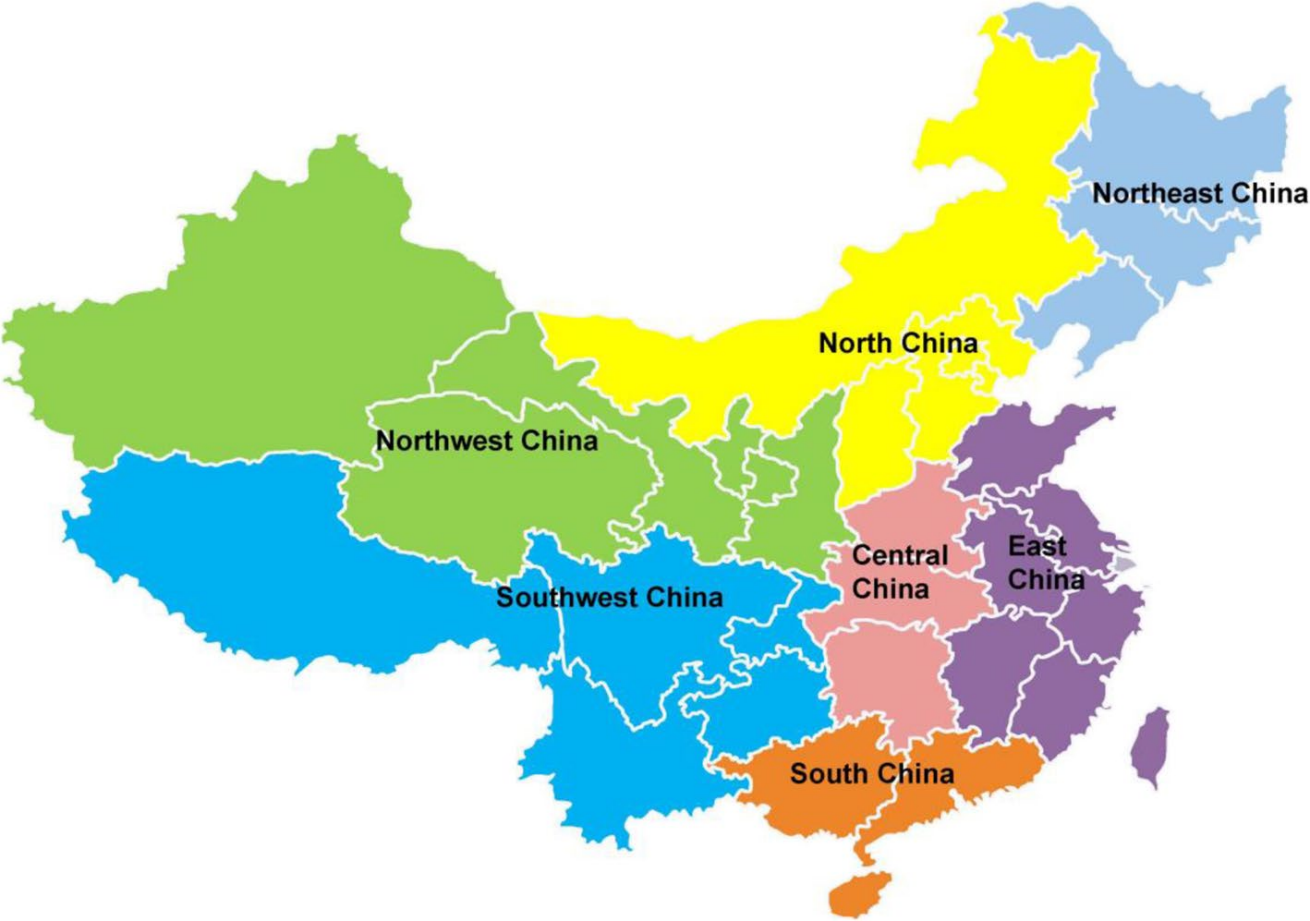
Distribution of the participants. Note: Different colors represent different geographic regions of China

The 15,332 participants’ geographic distribution was categorized into seven major geographic areas in China (see Fig. 1). The details of the total participants’ demographic characteristics can be found in Table 1, also with the female group and male group. The mean age of the participants was 32.9 ± 5.94 years old, and the population aged 31–40 accounted for the most (51.1%). The majority of them were first married (97.3%), Han nationality (74.6%). Notably, the number of persons living in the city was nearly 6 times that in the countryside. More than half of the participants had a bachelor’s degree (56.0%) and also the lowest monthly family income (54.5%). Most of the participants own house with loan (67.8%). Regarding occupation, the proportion of healthcare staff was the largest in total (71.0%) and female group (78.4%). Approximately four-fifths of them (79.7%) already had one or two existing children.

**Table 1:** Sociodemographic characteristics of participants (*N* = 15,332)

Characteristics	Total (***N*** = 15,332) n/%	Female (***N*** = 12,852) n/%	Male (***N*** = 2480) n/%
Age (years)	32.9 ± 5.94	32.8 ± 5.84	33.2 ± 6.44
Marital status			
First marriage	14,923/97.3	12,519/97.4	2404/96.9
Remarriage	409/2.7	333/2.6	76/3.1
Ethnic group			
Han nationality	11,434/74.6	9137/71.1	2297/92.6
Minority	3898/25.4	3715/28.9	183/7.4
Areas			
East China	8419/54.9	6410/49.9	2009/81.0
South China	3902/25.5	3709/28.9	193/7.8
Northwest China	1599/10.4	1523/11.9	76/3.1
Central China	692/4.5	634/4.9	58/2.3
Southwest China	343/2.2	321/2.5	22/0.9
North China	253/1.7	175/1.4	78/3.1
Northeast China	124/0.8	80/0.6	44/1.8
Residence			
city	13,029/85.0	10,937/85.1	2092/84.4
rural	2303/15.0	1915/14.9	388/15.6
Education			
High school	247/1.6	177/1.4	70/2.8
College	5230/34.1	4350/33.8	880/35.5
Bachelor	8582/56.0	7418/57.7	1164/46.9
Master	1022/6.7	732/5.7	290/11.7
Doctor	251/1.6	175/1.4	76/3.1
Occupation			
Healthcare staff	10,885/71.0	10,075/78.4	810/32.7
Worker, as teacher, civil servant, office worker	2585/16.9	1723/13.4	862/34.8
Laborer	1088/7.1	589/4.6	499/20.1
Individual operator	305/2.0	169/1.3	136/5.5
Farmer	102/0.7	71/0.6	31/1.3
Soldier	36/0.2	5/0.0	31/1.3
Freelancer	331/2.2	220/1.7	111/4.5
Family monthly income (¥)			
5000–10,000	8350/54.5	7190/55.9	1160/46.8
10,001–20,000	4728/30.8	3889/30.3	839/33.8
20,001–30,000	1426/9.3	1154/9.0	272/11.0
>30,000	828/5.4	619/4.8	209/8.4
House			
Tenancy	1924/12.5	1589/12.4	335/13.5
Housing with loan	10,389/67.8	8718/67.8	1671/67.4
Housing without loan	3019/19.7	2545/19.8	474/19.1
Number of existing children			
None	3106/20.3	2431/18.9	675/27.2
One	6660/43.4	5703/44.4	957/38.6
Two	5566/36.3	4718/36.7	848/34.2

### Third birth intention status

Among all the participants in this study, only 12.2% of them expressed a desire to have a third child. Most of the subjects (70.0%) reported that they had the same intentions as their spouses. More results about third birth intention of different groups can be found in Table 2.

**Table 2:** Third birth intention of total participants, female group and male group

Variables	Total (***N*** = 15,332) n/%	Female (***N*** = 12,852) n/%	Male (***N*** = 2480) n/%
Third birth intention			
very unintended	6910/45.1	5828/45.3	1082/43.6
unintended	6561/42.8	5616/43.7	945/38.1
intended	873/5.7	711/5.5	162/6.5
slightly intended	484/3.2	382/3.0	102/4.1
strongly intended	504/3.3	315/2.5	189/7.6
Third birth intention of spouse			
uncertain	3118/20.3	2681/20.9	437/17.6
Equal intention	10,736/70.0	8928/69.5	1808/72.9
Husband’s intention stronger	993/6.5	877/6.8	116/4.7
Wife’s intention stronger	485/3.2	366/2.8	119/4.8

### Reasons for different decision to have three children

For those who were willing to have three children, 22% wanted to have both a son and a daughter, 14.8% believed that a third child would be conducive to their children’s development, and 14.3% simply liked children.

For those who did not want three children, the leading reasons in order were a busy work schedule (29.2%), the high cost of raising and educating children (28.1%), and the impact of the concept of fewer births (10.6%). More details are shown in Table 3.

**Table 3:** Reasons of being intented or unintented to have three children

Classification	Category	Items	Response frequency (n/%)
Reasons for intention to have three children	Attitudes	Conducive to the growth of children	803/14.8
		Purely like children	772/14.3
		More children bring more blessings	496/9.2
		Reducing pension risks	414/7.6
		Enhancing couple’s relationship	406/7.5
	Subjective norm	Have both son and daughter	1189/22.0
		Elders’ expectations	559/10.3
		Husband/wife expectations	381/7.0
		Influence of surrounding peer groups	150/2.8
	Perceived control	Health status of existing children	133/2.5
		Increase family labor	112/2.1
Reasons for not wanting three children	Subjective norm	Husband / wife does not want	1167/2.7
		Impact of the concept of fewer births	4578/10.6
		The first or second child does not want	358/0.8
		Elders don ‘t want	412/1.0
	Perceived control	Older ages	4135/9.6
		More fertility impairs health	4135/9.6
		Pursuit personal career development	2139/5.0
		Fear of childbirth pain	1014/2.4
		Worried about body shape	454/1.1
		Busy at work, having no time and energy to take care of children	12,618/29.2
		High cost of upbringing and education of children	12,133/28.1

### Factors influencing third birth intention

The minimum tolerance was 0.650 (all larger than 0.1), and the maximum variance inflation factor (VIF) was 1.538 (all less than 10), which indicated that there was no notable multicollinearity problem. The *P* value of likelihood ratio test was less than 0.001, indicating that the model had adequate fit. The chi-square value of the Hosmer and Lemeshow test was 9.558, and the *P* value was 0.297 (greater than 0.05), indicating that the model had adequate fit. Many factors, including age, gender, ethnic group, marital status, residence, education, house ownership, monthly household income, and the number of existing children, were all influencing factors of third birth intention in the Chinese population of childbearing age (Table 4).

**Table 4:** Binary logistic regression analysis for factors related to third birth intention

Variables	***B***	***Wald χ2***	***P***	***OR***	***95%CI***
Age	−0.041	51.989	< 0.001	0.960**	[0.950,0.971]
Sex	0.792	154.329	< 0.001	2.209**	[1.949,2.503]
Ethnic group	0.598	103.357	< 0.001	1.819**	[1.612,2.042]
Marital status	0.850	46.513	< 0.001	2.340**	[1.833,2.988]
Education (ref: high school)					
Bachelor	−0.474	7.548	0.006	0.623*	[0.444,0.873]
Master	−0.591	8.149	0.004	0.554*	[0.369,0.831]
House (ref: tenancy)					
Housing with loan	− 0.173	4.785	0.029	0.841*	[0.720,0.982]
Family monthly income (¥) (ref: 5000–10,000)					
10,001–20,000	−0.217	11.166	0.001	0.805*	[0.709,0.914]
20,001–30,000	−0.246	5.015	0.025	0.782*	[0.630,0.970]
Residence	0.185	7.442	0.006	1.203*	[1.053,1.374]
Number of existing children (ref: none)					
One	−0.159	3.959	0.047	0.853*	[0.729,0.998]
Two	0.609	53.067	< 0.001	1.839**	[1.561,2.166]
Constant	−0.657	7.417	0.006	0.518**	

*OR* odds ratio, *CI* Confidence interval

^*^ *P* < 0.05

^**^ *P* < 0.001

## Discussion

### Low intention of the childbearing-age population to have three children in mainland China

Approximately one-eighth (12.2%) of respondents stated that they desired to have three children, which was lower than the second birth intention of China’s childbearing-age women (36.2%) and floating population (21.3%) [22, 23]. The perceived ideal number of children was 2, which was in line with the nationally representative cross-sectional Bangladesh Demographic and Health Survey 2014 data [24]. The third birth intention of most couples in this survey were the same (70.0%), which was consistent with the reports of Gibbs (92% perceived concordance with partner fertility desire) [25]. Undoubtedly, there was a certain bias in the reported birth intention of the other partner in the couple, which may be largely attributable to the perceptions of partners’ fertility preferences [26]. Birth intention is an important predictor of future population growth in a country. Reproductive decisions are made based on couples’ intentions, and the lack of consideration of partner intentions is a missed opportunity to better comprehend the couple-based nature of fertility behavior [27]. Recent studies in the United States corroborate this idea, showing that each partner influences the probability of having a child [28, 29]. Moreover, there is a strong link between baseline fertility desires and subsequent reproduction [30].

Fertility intention is not stable and changes as conditions change. Studies have shown that the decision to have children is multifaceted and determined not only by individual and economic factors but also by social policies [31, 32]. The results imply that policy makers and producers of reproductive support measures need to consider the fluidity of fertility desires when taking measures to encourage fertility. If conditions become favorable, desires to have three children could be stimulated and may lead to short birth intervals or eventually translate into fertility behavior.

Therefore, relying solely on policies to promote an increase in birth intention is not sufficient. We should shift our focus in policy making to understanding and guiding the third birth intention status of the childbearing-age population.

### Time and energy limitations and the high cost of education were major reasons for low intention

The upbringing, care and education of children are time and energy consuming. Regardless of the occupation type of the childbearing-age group, a busy work schedule, coupled with the parenting of multiple children, can exhaust parents. This is also the main reason why they do not want to have three children. Otherwise, in the Chinese context, due to the lack of public subsidized childcare services, market-based childcare has become the mainstream, leading to a substantial increase in the cost of childcare.

For school-age children’s education, the financial burden on the family has also increased due to the expenditure on education within and outside of school. The off-campus expenses accrue through parents registering for various supplementary and interest classes for their children. On-campus fees include the purchase of school district housing and the payment of school selection fees. According to data from the China Education Panel Survey (CEPS), the average household expenditure on extracurricular tutoring classes nationwide reached 2268 yuan per year in 2014 [33].

Therefore, increasing financial investment in public nursery services and reducing the education costs of children of school age will contribute to increasing the desire to bear more children and promote reproductive behavior.

### Sociodemographic characteristics differ in third birth intention

We found in this study that participants’ willingness to bear a third child gradually declined as age increased (*OR* = 0.960, *95% CI* = 0.950,0.971), which was not consistent with the findings of a fertility desire investigation in Bangladesh [24]. That study found that older couples desired more children than younger ones. It may be that older parental age is associated with reduced fertility and a perceived higher risk of pregnancy and neonatal complications [34]. This also suggests that if the government routinely provides preconception health promotion for older couples in primary medical institutions to help them feel that the risks of pregnancy are more controlled, their third-child fertility intentions may be improved.

The willingness of men to have three children was more than twice that of women. Odusina et al. examined couples’ fertility intentions and found that the husband’s willingness to have more children was 49.3%, which was higher than the wife’s willingness of 43.9% [35], similar to the results of this study. This was also consistent with the second birth intention of the floating population, in which women have significantly lower intentions than men [23]. This may be related to the diverse social responsibilities caused by gender differences. Regarding reproductive behavior, women bear the main duty of pregnancy and birth, which represents a larger risk to women’s physical and psychological health than to men’s. Moreover, for professional women, having more children means that job stability and career development face greater challenges and threats. Therefore, women are more cautious and conservative in the expression of third birth intention. Many studies gauging men’s fertility intention have indicated that men almost universally express a desire for parenthood, which could help sustain a genetic link to the child and is central to masculinity [36, 37]. Furthermore, men perceive fewer problems relating to balancing work and family life and are less concerned about combining work and parenthood [36]. Having a permanent position and having advanced in one’s profession are the only preconditions for parenthood among men, and they are less likely to believe that having another child will affect their status in the labor market [38].

The third birth intention of minority nationality is stronger than that of Han nationality (*OR* = 1.819, *95%CI* = 1.612–2.042), which is consistent with the research of second birth willingness of nurses in 6 tertiary hospitals in Urumqi [39]. It may be related to the long-term influence of the late marriage and 35-year long one-child policy on Han groups [40].

In previous studies, more attention has been paid to the differences in fertility intention between married and single persons [17], and less is known to the relationship between first or remarried marital status and third birth intention. We find that remarried participants are 2.34 times more likely to have the third birth intention by contrast of those in first marriage, which is consistent with the research of Wu (*OR* = 3.041, *95%CI* = 1.418–6.521) [41]. The potential causes may be related to the tendency to choose co-fertility of reorganized families, which could help sustain and strengthen the emotional relationship between couples and family members.

The analyses of the cross-sectional data on the relationship between education and fertility desire revealed a diversity of findings [15, 42–47]. Education is one of the most established socioeconomic determinants of fertility intentions [43] and is inversely associated with fertility behavior [44]. Previous studies in other developing countries emphasize that women’s fertility intentions tend to decline with educational attainment [45], especially for females in the floating population [46]. The results of this study are similar to the above research. Comparing to participants with high school degree, the third birth intention of those with a bachelor’s degree and a master’s degree decreased by 37.7% and 44.6%, respectively, which may be related to the phenomenon that improving education allows women to pursue their own careers and have freed some of them from raising children [47].

Housing type is associated with third birth intention according to the systematic review [17]. We also found that, even participants who rent a house now, their willingness to give birth the third child are 15.9% stronger than those who own house with loan (*OR* = 0.841, *95%CI* = 0.720–0.982). Compared with renting a house, the economic pressure of buying a house with loan in China is greater. Additionally, couples have to invest a high share of their economic resources into home ownership, which necessarily competes with the high costs of bearing another child [48]. If the loan interest rate can be lowered or more convenient and sustainable rental services could be provided, it will help increase intention of childbearing population to have more children in China context.

Annual household income has a positive effect on the rate of preferred birth: the higher the economic status is, the more attention given to the quality of childbearing and the greater the desire not to have another child. The third birth intention of those with monthly family income less than 20,000 and 30,000 yuan, is 80.5% and 78.2% of those with less than 10,000, respectively. In addition, the analysis of the reasoning for different third birth intentions indicated that the high costs of raising and educating children were the second major reasons for unwillingness to have a third child. With the continuous development of China’s social economy and the catalytic effect of family planning on family transformation, families have begun to follow modern reproductive trends. Meaningfully, in addition to the basic living security of children, families focus on children’s emotional wellbeing and quality education. Thus, parents are willing to pay more money and spend more energy to cultivate their children, with fertility costs becoming higher. This is consistent with Becker’s child quantity and quality substitution theory [49]. The important implication of this theory is a negative correlation between the quantity and quality of children, which means that raising the quality of child care may lead to a decline in the number of children. The relationship between economic income and fertility intention is also applicable to some countries in Africa. Additionally, for economic reasons, people of childbearing age experience the constant dilemma of the high fertility costs of additional children and the desire for a larger family [50]. Previous studies stated that persons may revise their preferences if their financial circumstances improve [30].

Historically, fertility decreases started earlier and developed faster in cities than in rural regions. In the present study, the third birth intention of the rural population was 1.203 times that of the urban population, which was consistent with the results from the 2012 Niger Demographic and Health Survey (*OR* = 1.61, *95% CI =* 1.20-2.17) [51]. Previous studies have also shown that the realization of fertility intention in rural areas is significantly higher than that in urban areas [52, 53]. The reasons may be related to the different regional contexts and fertility ideologies [54], such as overall maternal employment, the high cost of living, and enhanced educational and labor market opportunities in the urban context. All of the above factors have become barriers to the increase and realization of birth intentions.

Compared with those who have not yet had living children, the third birth intention of those who already have two children increases 83.9%. However, those who have only one existing child are less willing to have the third child (*OR* = 0.853, *95% CI* = 0.729–0.998). Some disparities with studies conducted in sub-Saharan Africa exist in this regard, as one study found that those who had four or more living children were less likely to desire more children (*OR* = 0.09, *95%CI* = 0.07–0.12) [55]. This may be related to the different populations investigated, differences in cultural context and the variance in the number of existing children. Another study also indicated that among childless women in the US, those who experienced infertility and were identified as infertile reported the highest ideal number of children (M = 2.35) [56]. However, this study did not include the previous experience of infertility as an independent variable. Therefore, whether this conclusion is applicable to the Chinese population needs to be further verified.

### Limitations

Three limitations of this study deserve mention. First, the imbalance in the distribution of the population was mainly manifested in the concentration of the population in certain geographical areas. Participants in East China accounted for the highest proportion, exceeding 50% of the total population. A previous study also pointed out that fertility intentions may vary across regions [23], which was not considered in this research. In addition to the unbalanced geographical distribution, selection bias cannot be ignored in terms of occupation, with healthcare workers enjoying the highest proportion (71.0%). In addition, this study used WeChat to disseminate the questionnaire. Participants without access to this social media application were probably not included, and the response bias could not be estimated. Finally, the third birth intention of spouse was limited to the perception of partner’s desire, which may lead to a moderate agreement of the intention between spouse [57].

## Conclusion

The general level of the third birth intention of the Chinese childbearing-age population is not high. Exploring the sociodemographic characteristics that affect third birth intention is helpful to understand the fertility desire of different groups and provide clues for exploring potential fertility drivers. The childbearing age of populations who are younger, male, minority, remarriage, with lower education and family monthly income, living in rural and already having two children show higher level of third birth intention. The results imply that effective strategies to stimulate the third birth intention of the reproductive-age population should be implemented and such strategies should allow people to combine paid work with parental responsibility, including through flexible working hours, part-time work, unpaid parental leave and affordable housing and childcare guarantees. This would be especially meaningful to improve the birth intention of individuals with high-quality resources, like higher level of education and economic resources.

## Supplementary Information

**Additional file 1.**

## Data Availability

The datasets used and/or analysed during the current study are available from the corresponding author on reasonable request.

## Abbreviations

CI: Confidence interval
OR: Odd ratio
