# Supplementary material for: Third birth intention of the childbearing-age population in mainland China and sociodemographic differences: a cross-sectional survey
Source: BMC Public Health. 2021 Dec 14;21:2280. doi: 10.1186/s12889-021-12338-8 (PMC8670058; doi:10.1186/s12889-021-12338-8)
Supplement: Supplementary file 1 — Additional file 1. [file 12889_2021_12338_MOESM1_ESM.pdf]

三孩生育政策背景下育龄人群生育意愿研究

尊敬的女士/先生：

您好！我们是青岛大学附属医院的研究团队，为了解三孩政策后育龄人群的生育意愿，特组织本次调查，调查对象为育龄人群（已婚人员，年龄20～45岁，尚未怀三胎）。调查仅用于学术研究，匿名填写，绝不透露个人隐私，请您根据实际情况放心填写。

您的意见对我们非常重要，感谢参与！

一、基本信息

1.您的性别：（1）男 （2）女

2.您的年龄：___岁

3.民族：（1）汉族 （2）少数民族

4.您的所在地：_____省_____市_____区

5.您的受教育程度：

（1）高中、中专、职高或技校（2）大专（3）大学本科（4）硕士（5）博士及以上

6.您的职业：

（1）医务工作者（2）职工：公务员/事业单位员工/教师等（3）工人（4）农民（5）军人（6）个体经营者（7）自由职业者

7.您的家庭月收入大概：___元/月

（1）1万元以下 （2）1万-2万 （3）2万-3万 （4）3万及以上

8.您的居住地为：

（1）城市 （4）农村

9.您家庭的住房情况：

（1）租房（无房） （2）有房，有贷款 （3）有房，无贷款

10.您的婚姻状况：

（1）一婚（2）再婚

11.您现有子女数量：

（1）0个 （2）1个 （3）2个或以上

二、生育意愿及原因调查

1.您是否有生育“三孩”的意愿？

（1）非常有意愿 （2）稍微有意愿 （3）有意愿（4）无意愿 （5）非常无意愿

1a.如果选择的是1、2、3 ，请勾出三项想生三孩的原因

☐利于一孩及二孩成长 ☐希望儿女双全 ☐纯粹喜欢孩子

☐增进夫妻感情 ☐长辈的期望 ☐丈夫/妻子的期望

☐降低养老风险 ☐周围同辈群体的影响

☐多子多福等传统观念的影响 ☐增加家庭劳动力

☐一孩/二孩身体健康状况

1b.如果选择的是4、5，请勾出三项不想生三孩原因：

☐经济压力（抚养、教育成本高） ☐没有时间精力照顾（工作忙）

☐少生观念影响(两个已经足够了) ☐长辈不希望 ☐丈夫不希望

☐一孩/二孩不希望 ☐夫妻感情原因 ☐害怕分娩痛

☐生理健康压力（包括年龄大） ☐个人事业发展考虑 ☐担心身材走样

☐周围同辈群体的影响

2.您期望子女数量：（ ）

（1）0个 （2）1个 （3）2个 （4）3个或以上

3.您期望子女数量及性别：___个女孩，___个男孩
4.您的三孩生育计划是
（1）1 年内 （2）2年内 （3）3～5 年内 （4）暂时不明确
5.您认为您丈夫/妻子的三孩生育意愿如何?
（1）平等意愿（2）丈夫意愿更强（3）妻子意愿更强 （4）不确定
6.“三孩政策”对您生育意愿的影响程度？（ ）
（1）完全不影响 （2）不太有影响（3）有些影响 （4）影响非常大

English version

Research on third birth intention of the childbearing-age population in China after the issue of three-child Policy

Dear Madam/Sir,

Hello! We are a research team from The Affiliated Hospital of Qingdao University. In order to understand the fertility intention of childbearing-age population after the three-child policy, we specially organized this large sample study. Individuals who are married, 20-45 years old, and not pregnant with the third child at the time of survey are all welcome to participate. The result of the research is only for academic publication, and all the answers in questionnaires are anonymous without disclosing personal privacy. Please feel free to fill carefully, according to the actual situation.

Your opinion is very important to us, thank you for your participation!

First part: Sociodemographic characteristics

1. Sex (1)male (2)female
2.Age _______years old
3.Ethinic group (1) Han nationality (2)Minority nationality
4.Area you live, Province_______
5.Your education level (1)High school (2)college (3)Bachelor (4)Master (5)Doctor
6.Please choose your occupation in the following.
(1) Healthcare staff, doctor, nurse or others caring for patients
(2) Worker, like teacher, civil servant, office worker, et al
(3) Laborer (4) Individual operator (5) Farmer (6) Soldier (7) Freelancer
7.What is your monthly household income level?
(1) less than 10000 yuan
(2) more than 10000 yuan and less than 20000 yuan
(3) more than 20000 yuan and less than 30000 yuan
(4) more than 30000 yuan
8. what’s your residence in recent years, city or rural?
(1) City (2) Rural
9. Do you or your spouse own a house?

(1) No, living in renting house
(2) Own house with loan
(3) Own house without loan
10. what's your marriage status?
(1) First married   (2) Remarried
11. How many children do you have now?
(1) None (2) One existing child (2) Two existing children

Second part: Fertility intention questionnaires

1.Do you intend to have three children?
(1) very unintended
(2) unintended
(3) intended
(4) slightly intended
(5) strongly intended

1a. If you choose option (1), (2), (3), please tick reasons bellow why you want to have three children?

1) Conducive to the growth of children
2) Purely like children
3) More children bring more blessings
4) Reducing pension risks
5) Enhancing couple's relationship
6) Have both son and daughter
7) Elders' expectations
8) Husband/wife expectations
9) Influence of surrounding peer groups
10) Health status of existing children
11) Increase family labor

1b. If you choose option (4), (5), please tick reasons bellow why you don't want to have three children?

1) Husband / wife does not want
2) Impact of the concept of fewer births
3) The first or second child does not want
4) Elders don 't want
5) Older ages
6) More fertility impairs health
7) Pursuit personal career development
8) Fear of childbirth pain
9) Worried about body shape
10) Busy at work, having no time and energy to take care of children
11) High cost of upbringing and education of children

2. Your ideal number of children
(1) none (2) one (4) two (5) three or more
3.The gender structure of your ideal number of children, ____boy(s), ____girl(s)

4. When will you plan to have the third child?
(1) within 1 year
(2) within 2 years
(3) within 3-5 years
(4) uncertain
5. As far as you know, what is your husband's or wife's intention to have three children?
(1) Uncertain
(2) Equal intention
(3) Husband’s intention stronger
(4) Wife’s intention stronger
6.How much does the ‘three-child policy’ affect your intention to give birth three children?’
(1) absolutely not
(2) little
(3) some
(4) absolutely yes
